# Extinct Beringian wolf morphotype found in the continental U.S. has implications for wolf migration and evolution

**DOI:** 10.1002/ece3.2141

**Published:** 2016-04-24

**Authors:** Julie A. Meachen, Alexandria L. Brannick, Trent J. Fry

**Affiliations:** ^1^ Anatomy DepartmentDes Moines University3200 Grand AveDes MoinesIowa50312; ^2^ Department of BiologyUniversity of WashingtonBox 351800SeattleWashington98195

**Keywords:** Canis, last glacial maximum, migration, wolf

## Abstract

Pleistocene diversity was much higher than today, for example there were three distinct wolf morphotypes (dire, gray, Beringian) in North America versus one today (gray). Previous fossil evidence suggested that these three groups overlapped ecologically, but split the landscape geographically. The Natural Trap Cave (NTC) fossil site in Wyoming, USA is an ideally placed late Pleistocene site to study the geographical movement of species from northern to middle North America before, during, and after the last glacial maximum. Until now, it has been unclear what type of wolf was present at NTC. We analyzed morphometrics of three wolf groups (dire, extant North American gray, Alaskan Beringian) to determine which wolves were present at NTC and what this indicates about wolf diversity and migration in Pleistocene North America. Results show NTC wolves group with Alaskan Beringian wolves. This provides the first morphological evidence for Beringian wolves in mid‐continental North America. Their location at NTC and their radiocarbon ages suggest that they followed a temporary channel through the glaciers. Results suggest high levels of competition and diversity in Pleistocene North American wolves. The presence of mid‐continental Beringian morphotypes adds important data for untangling the history of immigration and evolution of *Canis* in North America.

## Introduction

The late Pleistocene fauna in North America was different and more diverse than it is today. Mammals were found in greater species diversities, population numbers, and body sizes (Smith et al. 2003). The dire wolf (*Canis dirus*) was an iconic canid species that is often associated with the Ice Age, and unlike its close relative the gray wolf (*Canis lupus*) which came from Eurasia, the dire wolf arose in North America and is endemic to the Americas (Wang and Tedford 2008; Tedford et al. 2009). When dire wolves went extinct at the end of the Pleistocene epoch (circa 11,500 years ago), it enabled the gray wolf to move into the territory that is now mid‐continent North America in much larger numbers (Dundas 1999). Interestingly, the dire wolf is present, but not commonly found above 42°N latitude (Neotoma and PBDB). Gray wolves are well‐documented north of these latitudes, well into the Arctic Circle, in both the late Pleistocene and the Holocene (www.neotomadb.org). However, gray wolves are found in much lower densities in the late Pleistocene of North America. For example, at Rancho La Brea in southern California dire wolf fossils outnumber gray wolf fossils by a factor of more than five (A. Farrell, pers. comm. – Rancho la Brea collection internal database).

Movement of these two canid species in North America may seem simple upon a cursory examination, but in depth studies of *Canis* genetics and morphology present a much more complicated ecological and evolutionary picture. Genetic diversity in *Canis* has been well‐studied in North America (vonHoldt et al. 2008, 2010, 2011; Koblmuller et al. 2009; Munoz‐Fuentes et al. 2009; Wayne and Hedrick 2011; Fredrickson et al. 2015; Hendricks et al. 2015), but extant wolf genetic variation was not very well‐understood until recently (vonHoldt et al. 2011; Mech et al. 2014; Cronin et al. 2015) and Pleistocene *Canis* genetics and morphology show even greater variation than what has been seen in Holocene populations (Leonard et al. 2007; Koblmuller et al. 2012; Meachen and Samuels 2012; Meachen et al. 2015; Pardi and Smith 2015).

In 2007, fossils of a previously unrecognized, extinct *Canis lupus* ecomorph were described from Alaska called Beringian wolves (Leonard et al. 2007). It was not specified if this new wolf group was deserving of a subspecies designation, but they did show that the Beringian wolves were unique, both morphologically and genetically, and considered to be well‐suited to the cold, megafauna‐rich environment of the late Pleistocene and the last glacial maximum (LGM). Beringian wolves went extinct at the end of the Pleistocene along with the dire wolf and numerous other megafaunal species (Leonard et al. 2007). Beringian wolves converged on dire wolf morphology with robust jaws, large, broad carnassial teeth, and wide, short snouts – all modifications for higher bite force and better carcass processing (Leonard et al. 2007). Beringian wolves also show increased levels of tooth breakage and wear compared with living gray wolves (Leonard et al. 2007), similar to levels seen in dire wolves from Rancho La Brea (Van Valkenburgh and Hertel 1993; Binder et al. 2002; Binder and Van Valkenburgh 2010). This suggests that the Beringian ecomorph experienced elevated levels of competition compared to current gray wolf populations and/or processed carcasses more fully than its modern counterparts. These convergent traits show niche overlap in Beringian and dire wolves, but geographic separation with dire wolves in mid‐ to southern latitudes and Beringian wolves in northern latitudes.

**Figure 1:**
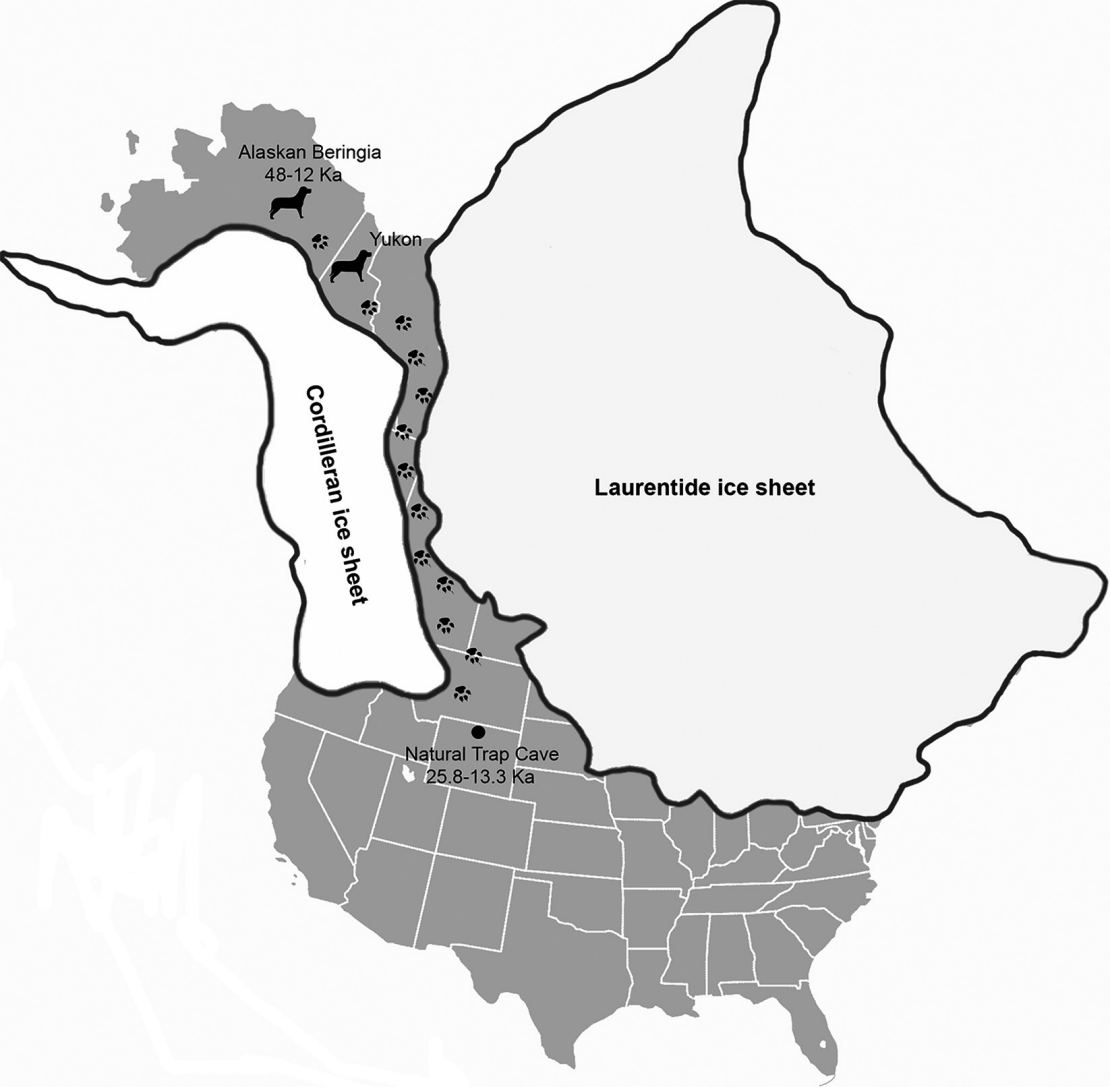
Map of North America with Pleistocene glaciers and the assumed path of Beringian wolves from Alaska to NTC. Dog icons represent sites where Beringian wolves have previously been found and paw prints represent the hypothesized path of the Beringian wolves through the Cordilleran and Laurentide ice sheets. Natural Trap Cave is denoted with a black dot.

The northern Wyoming fossil site of Natural Trap Cave (NTC) may hold some clues to Pleistocene wolf evolution and biogeography. This site is situated at the base of the Bighorn Mountain range, in the middle of the North American continent, an ideal location for examining north to south migrations of megafauna in the late Pleistocene (Martin and Klein 1984), since the location of this site is directly south of the split between the Laurentide and Cordilleran ice sheets and prior studies have shown migrations of Beringian bison from Alaska to NTC (Shapiro et al. 2004) (Fig. 1). Not only is this site geographically advantageous, but it is also unique and unusual because of the abundance of Pleistocene mammal fossils and its fossil preservation. The cave environment helps to protect the fossils by keeping temperature (40°F) and humidity (≈90%) relatively constant and by shielding the fossils from trampling or extreme weathering (Wang and Martin 1993). The large mammals that are found in the NTC fauna are mostly cursorial, arid, or open habitat dwellers. The most abundant species include at least two undetermined species of the genus *Equus*, a stilt‐legged horse and a caballine‐morph horse (Spencer and Scott 2013), the American cheetah‐like cat (*Miracinonyx trumani*), bighorn sheep (*Ovis canadensis*), and a wolf/wolves (*Canis* sp.) – the most common predator (Wang 1988; Wang and Martin 1993). Previously reported radiocarbon dates from the site suggest the mammal faunas span much of the LGM of Marine Isotope Stage ≈ 26,000–19,000 years before present (Clark et al. 2009; Kohn and McKay 2012). Specifically, the wolves at NTC date from 25,800 to 14,300 years before present (Kohn and McKay 2012).

*Canis* from Natural Trap Cave has been enigmatic for the last 30 years. It is unclear from the literature whether dire wolves, gray wolves, or some other canid is present at NTC (Martin and Gilbert 1978; Martin and Klein 1984; Wang and Martin 1993). If dire wolves are present at NTC, then it is the northernmost record of the species known to date (http://www.neotomadb.org; www.pbdb.org). The gray wolf also existed in mid‐continental North America during the late Pleistocene (http://www.neotomadb.org; www.pbdb.org); albeit at lower densities so it is possible it was common at NTC. If the recently recognized Beringian wolf ecomorph is present at NTC, then it is the first record of this morphotype in the continental United States.

A rigorous statistical analysis of morphological characters is needed in order to place these wolves into their correct typology. Here, we aim to statistically assess what species of *Canis* is/are present at NTC, what their morphology can tell us about their environment and ecological niche at this site, and what implications this has for the movement and location of different large canids in late Pleistocene North America.

## Methods

We examined four separate groups of *Canis* (full species lists and locations can be found in Table S1). The first group consisted of dire wolves from seven different asphalt pits at Rancho La Brea that vary in radiocarbon ages from approximately 38,000 to 13,000 years before present (*n* = 310) housed at the George C. Page La Brea Tar Pits Museum, and the University of California, Berkeley Museum of Paleontology. We originally separated each time interval of dire wolves into its own group following Brannick et al. (2015), but after an initial assessment of the data, we combined all dire wolves into one group, as they clustered together in this analysis. The second group was made up of Beringian wolves from Alaska belonging to a time interval of approximately 48,000–12,000 years before present (*n* = 21) housed at the American Museum of Natural History (AMNH), many of these were the same specimens that were haplotyped in Leonard et al. (2007). The third group was made up of extant populations of gray wolves from northern to mid‐continent North America (including specimens from Alaska USA, Minnesota USA, Montana USA, Oklahoma USA, Wyoming USA, Alberta CAN, Nunavut Territory CAN, and Northwest Territory and, CAN) housed at the AMNH (*n* = 33 total). The fourth group consisted of the *Canis* specimens from NTC housed in the Kansas Museum of Natural History collections (*n* = 7). NTC wolves currently have eight radiocarbon dates that place them between 25,800 and 14,300 years before present (Kohn and McKay 2012). For the purposes of this study we only examined individuals with whole mandibles and fully erupted permanent dentition. For modern specimens of known sex we attempted to include equal numbers of males and females. Sex data are unknown for fossil specimens.

**Figure 2:**
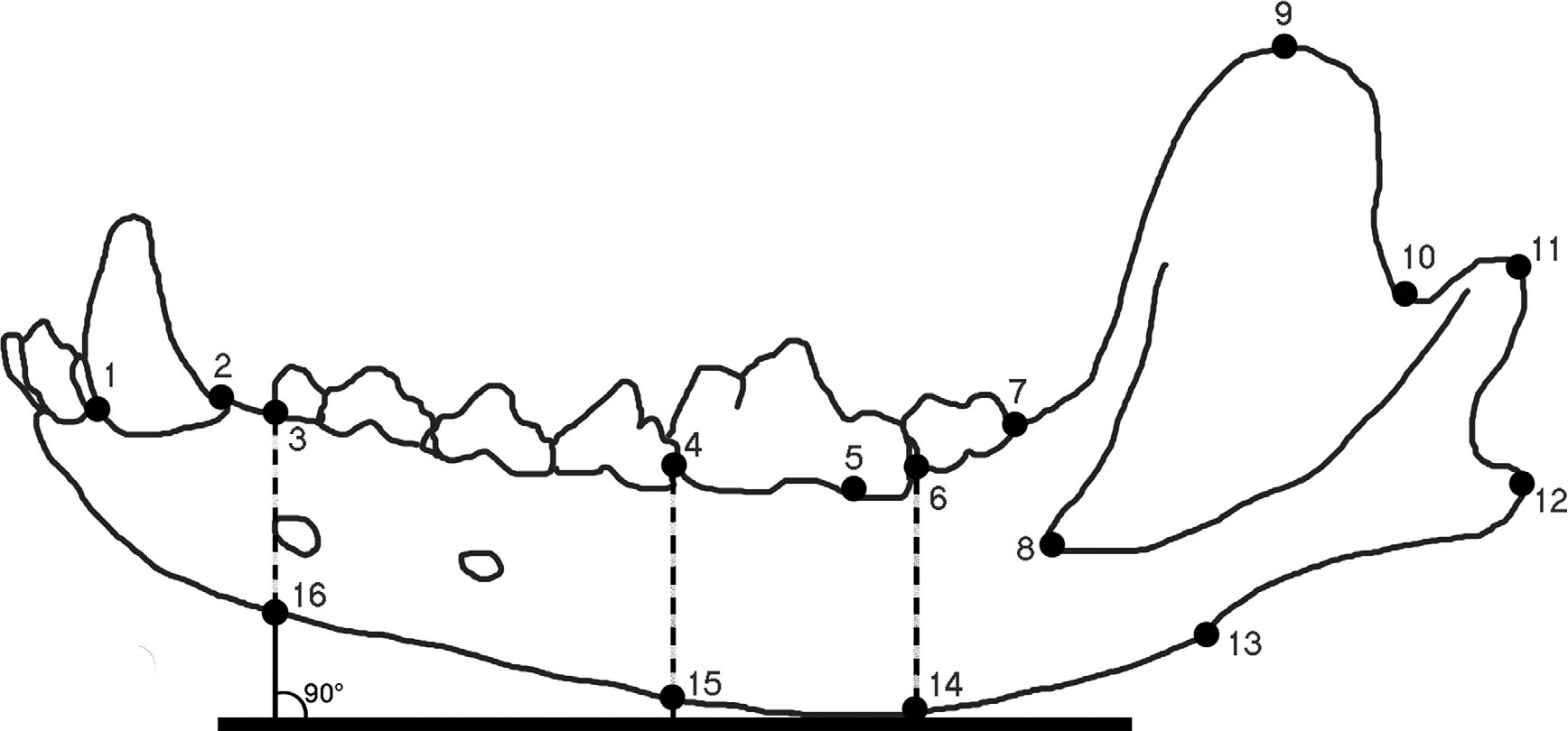
A wolf mandible with the 16 landmarks used in this study. Landmarks 14, 15, and 16 demonstrate how a straight edge and a 90° angle were used to place landmarks that fall directly below landmarks 6, 4, and 3 respectively.

We analyzed 2D geometric morphometric (GM) data from the mandibles on the different groups of wolves in this study. GM data is useful because it removes all but the allometric component of size, so analyses can focus on shape differences. Photographs of the labial side of each mandible were taken using a copy stand camera set‐up and utilizing sand boxes or modelling clay to position the mandible as flat as possible for the photograph. All other photography protocols, including those to minimize parallax, followed those in Meachen et al. (2014, 2015). Sixteen digital landmarks were placed on all mandibles in functionally significant, reproducible positions using the tpsDig2 program (version 2.17) (Rohlf 2013) (Table 1, Fig. 2). Although GM techniques focus on shape differences, we also collected scalar data by including a scale bar in every specimen photo at the same height as the mandible and using the “measure” tool in tpsDig2. These data were then used to compute the centroid size of each specimen, which is used to incorporate a measure of size. All sets of landmark coordinates were aligned using Procrustes least‐squares and the *x*,* y*‐coordinates were used to obtain a consensus configuration. We generated partial warp scores (localized shape differences) by comparing individual landmarks to the mean configuration (Zelditch et al. 2012). We analyzed all 2D GM data using the free programs in IMP8 (Sheets 2014). To see how well each group was separated in morphospace and how well each specimen was assigned to its group, we ran a canonical variates analysis (CVA) on the Procrustes coordinates using the program IMP:CVAGen8 (Sheets 2014).

**Table 1:** Landmarks placed on all *Canis* mandibles analyzed in this study

Landmark	Description
1	Anterior edge of the canine tooth at the tooth/mandible junction
2	Posterior edge of the canine tooth at the tooth/mandible junction
3	Anterior edge of the p1 at the tooth/mandible junction
4	Posterior edge of the p4 at the tooth/mandible junction
5	Point where the talonid basin (grinding surface) begins at the tooth/mandible junction, can be estimated at roughly 2/3 the length of the m1 (carnassial)
6	Posterior edge of the m1 at the tooth/mandible junction
7	Posterior edge of the m2 or m3 (last grinding tooth) or alveolus at the tooth/mandible junction
8	Innermost point of the masseteric fossa
9	Top‐most point of the coronoid process
10	Basin of the mandibular notch, used in conjunction with coronoid process to measure coronoid height
11	Posterior most point of the mandible at the condyloid process
12	Tip of the angular process
13	The end of the curve where the angular process transitions to the mandibular ramus. Most mandibles have a masseteric rugosity here that serves as a point of reference
14	Bottom edge of mandible directly below landmark 6, measured with a straight edge and a 90° angle (see Fig. 2)
15	Bottom edge of mandible directly below landmark 5, measured with a straight edge and a 90° angle
16	Bottom edge of mandible directly below landmark 4, measured with a straight edge and a 90° angle

A principal component analysis (PCA) was run on the covariance matrix of the Procrustes coordinates using the free program IMP:PCAGen8 (Sheets 2014) to determine which shape features were separating each group. We then ran a multivariate analysis of variance (MANOVA) on the PCA scores using Scheffé's and Tamhane's post hoc procedures for equal and unequal variances, respectively, in the program SPSS v.22 (IBM, 2013) to look for statistically significant differences between each wolf group. We ran a homogeneity of variance test to determine which post hoc procedure was appropriate.

## Results

**Figure 3:**
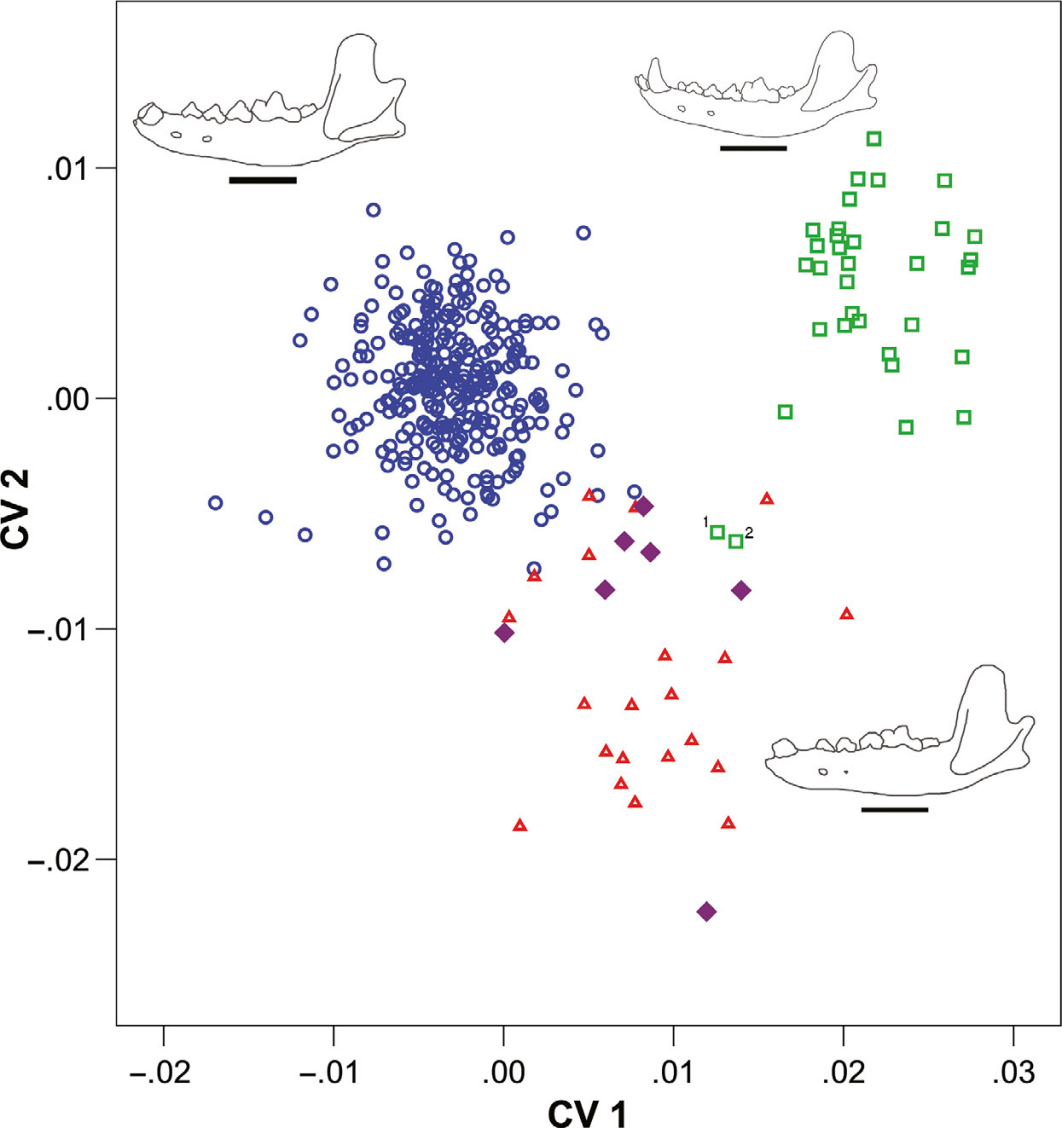
Canonical variates analysis showing morphogroups in CV1 versus CV2. Circles represent dire wolves, squares represent extant gray wolves, triangles represent Alaskan Beringian wolves, and filled diamonds represent NTC wolves. Outlines represent actual mandibles of extant gray wolves (top right), dire wolves (top left), and Alaskan Beringian wolves (bottom right). Scale bars are 5 cm. The two outlier modern gray wolf specimens represent: (1) AMNH 19,348 from Nunavut Territory, Canada; and (2) AMNH 2384 from Oklahoma, USA.

The canonical variates analysis yielded four axes, and CV1 and CV2 had eigenvalues >1 (axis 1–4.412; axis 2–1.31). The CVA showed three distinct morphogroups (Wilks' lambda 0.0636, *P *<* *0.001) (Fig. 3), with 94.8% correct classification and 93.8% correct jack‐knifed cross‐validation. Extant wolves have positive loadings on both CV1 and CV2, whereas dire wolves have negative loadings on CV1 and positive loadings on CV2. Beringian and NTC wolves group together with negative loadings on CV2 and positive loadings on CV1. In both PC and CV plots, NTC wolves fall between dire wolves and Beringian wolves, although they show a greater affinity toward Beringian wolves than dire wolves.

**Figure 4:**
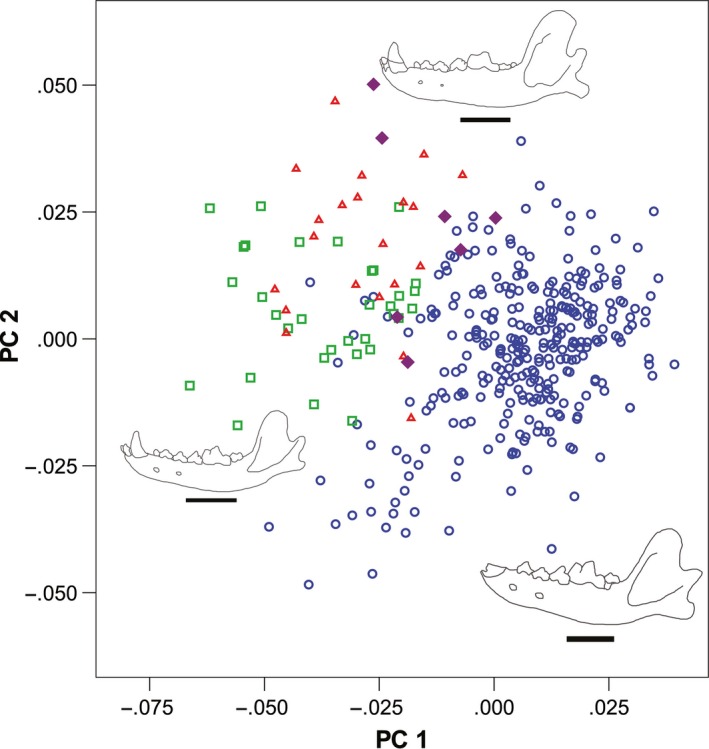
Principal components analysis showing PC1 versus PC2; symbol legend as in Fig. 3. Outlines represent actual mandibles of NTC wolves (top), extant gray wolves (bottom left) and dire wolves (bottom right). Scale bars are 5 cm.

The PCA yielded 28 principal components (PC), all with eigenvalues <1. The first three components account for 50% of the variation in the dataset. These three PCs showed differences between the four wolf groups. The remaining components highlighted individual specimen differences. The results showed separation among three groups (Fig. 4), with minor separations between NTC and Beringian wolves on PC3. PC 1 showed a separation of dire wolves from all other groups. The dire wolves had positive loadings that were associated with high coronoid processes, longer mandibles, deeper jaws (dorsoventrally), and shorter grinding areas of the lower m1, m2, and m3 (this tooth was absent in most of the dire wolf specimens). All other groups had more negative values on PC1.

On PC2 Beringian and NTC wolves grouped together with positive values, separate from all others. These positive loadings reflected a slightly longer, posteriorly reflected coronoid process, dorsoventrally deep jaws, a lower condyloid process and anteroposteriorly large lower canines. Dire wolves were scatted throughout this axis while extant wolves had neutral loadings at zero.

PC 3 was harder to interpret, but the positive values indicated higher coronoid processes and dorsoventrally shallower mandibles. NTC and extant wolves are found at the zero point (0,0) for PC3, while dire wolves are scattered throughout this axis. Alaskan Beringian wolves show negative loadings on this axis.

Results of the MANOVA on the principal component scores show that NTC wolves and Beringian wolves were statistically indistinguishable for PCs 1 and 2 (Table 2). On PC1, NTC wolves were significantly different from all groups except Beringian. On PC2, NTC wolves were not significantly different from Beringian, and did not show a strict significant difference from extant wolves (*P *=* *0.052), however, this low *P*‐value shows that these two groups have clear differences which may have been better elucidated with a larger sample size. For PC3, Beringian wolves were the only group that showed any significant differences in PC scores. In the analysis of centroid size dire wolves were significantly larger than all other groups. Beringian wolves were indistinguishable from NTC wolves, but were significantly larger than extant gray wolves. NTC wolves were also larger than extant gray wolves, but not significantly so, likely due to a small sample size (centroid size, mean ± SD: Dire wolves 304.5 ± 17.7; gray 242.8 ± 12.9; Beringian 258.3 ± 9.5; NTC 257.1 ± 18.0) (see Table 2 for *P*‐values).

**Table 2:** Results of the MANOVA run on the Procrustes principal component scores and centroid size (CS), significance at the *α *= 0.05 level is in bold

Variable	Comparison groups	*P*‐value
PC1	NTC versus dire wolves	**0.003**
	NTC versus extant	**0.010**
	NTC versus Beringian	0.265
	Dire wolves versus extant	**<0.001**
	Dire wolves versus Beringian	**<0.001**
	Beringian versus extant	0.281
PC2	NTC versus dire wolves	**<0.001**
	NTC versus extant	0.052
	NTC versus Beringian	0.955
	Dire wolves versus extant	**0.017**
	Dire wolves versus Beringian	**<0.001**
	Beringian versus extant	**0.014**
PC3	NTC versus dire wolves	1.000
	NTC versus extant	0.999
	NTC versus Beringian	0.120
	Dire wolves versus extant	0.978
	Dire wolves versus Beringian	**<0.001**
	Beringian versus extant	**0.001**
CS	NTC versus dire wolves	**0.002**
	NTC versus extant	0.409
	NTC versus Beringian	1.000
	Dire wolves versus extant	**<0.001**
	Dire wolves versus Beringian	**<0.001**
	Beringian versus extant	**<0.001**

## Discussion

Our results demonstrate that NTC wolves are neither dire wolves nor extant gray wolves. They are significantly distinct from dire wolves in both shape and centroid size. NTC wolves are also significantly morphologically distinct from extant wolf populations, including those that live in the area today. The wolves found at NTC closely align morphologically with the Pleistocene Beringian wolf populations from Alaska. This result is interesting for a number of reasons. First, this illuminates why previous researchers had trouble pinpointing exactly what type of wolves were at NTC. This morphotype falls somewhere between modern gray wolves and dire wolves in size and robustness, making it difficult to determine its assignment without a thorough statistical assessment. More importantly, the NTC population is the first known population of Beringian wolf ecotypes in mid‐continental North American and the southern‐most known occurrence as well. This has implications for pre‐Pleistocene extinction diversity of *Canis* throughout North America and dispersal patterns of late‐ and post‐Pleistocene *Canis*.

Our results show that there were at least three large *Canis* morphotypes in mid‐continent North America during the late Pleistocene, with some evidence of competitive exclusion in these three groups. The larger Beringian wolves are now documented mid‐continent, along with Pleistocene modern wolf morphotypes in California (and other U.S. states) and dire wolves from most of the mid‐continental U.S. (Meachen and Samuels 2012). This analysis shows that Beringian wolves have adaptations for forceful mastication and processing of carcasses. They have deep jaws, especially anterior to the molars, and a reduced molar arcade relative to slicing teeth. These adaptations are similar to those in dire wolves, but our dire wolves are larger than Beringian wolves with more robust premolars.

Beringian wolves have been genetically documented as far east as the Yukon Territory, with all Pleistocene Yukon wolf specimens sampled by Leonard et al. (2007) showing the extinct Beringian haplotype, which is distinct from extant Alaskan and Canadian populations. This shows a migration pattern of these wolves between the Laurentide and Cordilleran ice sheets from Alaska to Canada and finally to the mid‐continental region of the U.S. (Fig. 1). This immigration would have occurred well before the relatively late Late Wisconsinan advance of the Laurentide ice sheet over the interior of western Canada [LGM 18,500–16,000 cal years before present (Lacelle et al. 2013)] and the northern Rocky Mountains great basin glaciers [LGM 20,500–17,2000 cal years before present (Laabs et al. 2013)] since Beringian wolves date as early as 25,800 years before present at NTC (Kohn and McKay 2012).

Using the pattern of Beringian wolf migration that we see through the ice sheets in northern North America, we predict that Pleistocene‐age Beringian wolves may also be found along the edge of the Cordilleran ice sheet in Oregon and Idaho and along the edge of the Laurentide ice sheet in South Dakota, Iowa, Illinois, Indiana and possibly even points east. Beringian wolves should also be found north of NTC in Montana along the inter‐ice corridor they traversed.

In addition to our evidence for Beringian wolves at NTC, there is a growing body of data which reveals the movement of large mammal populations, such as bison (*Bison* spp.), lions (*Panthera atrox*) and brown bears (*Ursus arctos*), between Beringia and more southerly latitudes of continental North America prior to the Late Wisconsinan advance of the Laurentide Ice sheet (Matheus et al. 2004; Shapiro et al. 2004; Barnett et al. 2009). This free exchange of populations between north and south was abruptly halted by the coalescence of the Laurentide and Cordilleran ice sheets during a brief period during the Last Glacial Maximum, ≈16,000–13,000 years before present (Lacelle et al. 2013). Given the chronology of mammal faunas represented at NTC largely dating to prior to the maximum Laurentide advance, it is not completely surprising to find wolf populations of Beringian origin in the mid‐continent.

This Beringian wolf migration may have been related to an important prey species of Beringian wolves: woodland muskoxen (genus *Bootherium*). Fox‐Dobbs et al. (2008) found stable isotope values in the bone collagen of pre‐LGM Alaskan Beringian wolves suggesting that *Bootherium* was an important prey species. *Bootherium bombifrons* is present in the NTC fauna, and may have been the impetus for the initial Beringian wolf migration. Additionally, *Bison* is known to have migrated from Alaska to NTC in the late Pleistocene, and would also have been a viable prey species for wolves (Shapiro et al. 2004).

Dire wolves are not commonly found north of California, and are especially rare in the late Pleistocene. According to the Paleobiology and Neotoma databases there are only five unconfirmed records of dire wolves above 42°N latitude: Fossil Lake, Oregon (late Pleistocene), American Falls Reservoir, Idaho (Sangamonian), Salamander Cave, South Dakota (Irvingtonian), and four closely grouped Irvingtonian sites in northern Nebraska. Several of these more northern sites where dire wolves are present are Irvingtonian, which is considered middle Pleistocene and was over by approximately 240,000 years ago. There are no confirmed records of *Canis lupus* in North America at that time. From these records it is unclear if dire wolves co‐existed at all with gray wolves during this time. The dearth of late Pleistocene dire wolves in more northerly latitudes begs the question of whether this species had range restrictions due to temperature, prey, habitat, etc. Since dire wolves are missing from these sites in the late Pleistocene, this region would have been prime territory for Beringian wolves expanding their range south along the glacier line.

Regardless of how far south Beringian wolves made it into mid‐continental North America, we know that they went extinct at the end of the Pleistocene along with the other megafauna. Extant wolf populations from the same regions today do not share genetic or morphological affinity with the Pleistocene Beringian wolves (Leonard et al. 2007), and here we support the morphological distinction. As there is good morphological and isotopic evidence that Beringian wolves were megafaunal prey specialists (Leonard et al. 2007; Fox‐Dobbs et al. 2008), their extinction at the end of the Pleistocene is unsurprising. The next important question is: which gray wolf populations moved into the mid‐continental U.S. region when Beringian wolves went extinct? Did populations of modern gray wolves disperse northward to geographically replace the Beringian populations? Morphologically, we know that the Pleistocene southern U.S. wolf populations were very similar to modern populations (Leonard et al. 2007; Meachen and Samuels 2012).

The next steps in this project include morphological analysis of other populations of North American Pleistocene gray wolves and genetic analyses of NTC wolves. Mitochondrial DNA has already been successfully extracted from several other NTC species (Shapiro et al. 2004; Barnett et al. 2005, 2009; Weinstock et al. 2005), so we are hopeful that wolf specimens may prove fruitful. This DNA will be compared with existing samples from Beringian wolves and extant wolf populations (Leonard et al. 2007; vonHoldt et al. 2008, 2010, 2011; Hendricks et al. 2015) to better understand the genetic makeup of the NTC wolves. Are the NTC wolves the same haplotype as the Alaskan Beringian wolves or is there morphological convergence or admixture?

## Conclusions

This study found the first record of the Beringian wolf morphotype in the continental United States, significantly extending its range further south. Our findings demonstrate that the story of the gray wolf invading in large numbers at the end of the Pleistocene is complex. The Beringian wolf morphotype had dispersed southward into the region that is now the mid‐continental U.S. from Alaska and Canada through an ice corridor prior to the maximum advance of the Laurentide ice sheet. This begs new questions about how early the Beringian wolves dispersed southward, how widespread they were in North America and what this can tell us about the timing of their extinction and the immigration of the extant gray wolf. This study is another case, along with several recent morphological and molecular studies, showing us that sub‐specific extinctions happened across many still extant species at the end of the Pleistocene epoch (Wisely et al. 2008; Meachen and Samuels 2012; Matte et al. 2013). It also instantiates the complexity of *Canis* evolution and population dynamics over the past 50,000 years in North America.

## Conflict of Interest

None declared.

## Supporting information


**Table S1.** All *Canis* mandibles analysed in this study.
