# Supplementary material for: Extinct Beringian wolf morphotype found in the continental U.S. has implications for wolf migration and evolution
Source: Ecol Evol. 2016 Apr 24;6(10):3430–8. doi: 10.1002/ece3.2141 (PMC4870223; doi:10.1002/ece3.2141)
Supplement: Supplementary file 1 — Table S1. All Canis mandibles analysed in this study. [file ECE3-6-3430-s001.docx]

Table S1: All *Canis* mandibles analysed in this study

| Catalog number | Species | Locality | Age (Radiocarbon included if present) |
| --- | --- | --- | --- |
| AMNH 1551 | Canis lupus | Oklahoma | Recent |
| AMNH 2247 | Canis lupus | Montana | Recent |
| AMNH 2384 | Canis lupus | Oklahoma | Recent |
| AMNH 4362 | Canis lupus | No data | Recent |
| AMNH 5378 | Canis lupus | Oklahoma | Recent |
| AMNH 5380 | Canis lupus | Oklahoma | Recent |
| AMNH 10697 | Canis lupus | Wyoming, Big horn co | Recent |
| AMNH 10698 | Canis lupus | Wyoming, Big horn co | Recent |
| AMNH 16845 | Canis lupus | Alberta, CAN | Recent |
| AMNH 16849 | Canis lupus | Alberta, CAN | Recent |
| AMNH16851 | Canis lupus | Alberta, CAN | Recent |
| AMNH 19348 | Canis lupus | Nunavut Territory, CAN | Recent |
| AMNH 22940 | Canis lupus | Nunavut Territory, CAN | Recent |
| AMNH 29040 | Canis lupus | Northwest Territory, CAN | Recent |
| AMNH 31628 | Canis lupus | Alberta, CAN | Recent |
| AMNH 34446 | Canis lupus | Northwest Territory, CAN | Recent |
| AMNH 34956 | Canis lupus | Minnesota | Recent |
| AMNH 34958 | Canis lupus | Minnesota | Recent |
| AMNH 34961 | Canis lupus | Minnesota | Recent |
| AMNH 42119 | Canis lupus | No data | Recent |
| AMNH 98226 | Canis lupus | Alberta, CAN | Recent |
| AMNH 98227 | Canis lupus | Alberta, CAN | Recent |
| AMNH 98230 | Canis lupus | Alberta, CAN | Recent |
| AMNH 98232 | Canis lupus | Alberta, CAN | Recent |
| AMNH 121469 | Canis lupus | Northwest Territory, CAN | Recent |
| AMNH 121497 | Canis lupus | Northwest Territory, CAN | Recent |
| AMNH 130170 | Canis lupus | Alberta, CAN | Recent |
| AMNH 134940 | Canis lupus | Minnesota | Recent |
| AMNH 134941 | Canis lupus | Minnesota | Recent |
| AMNH 134942 | Canis lupus | Minnesota | Recent |
| AMNH 134943 | Canis lupus | Minnesota | Recent |
| AMNH 134944 | Canis lupus | No data | Recent |
| AMNH 169525 | Canis lupus | Alaska | Recent |
| AMNH 169526 | Canis lupus | Alaska | Recent |
| AMNH 169527 | Canis lupus | Alaska | Recent |
| AMNH 169528 | Canis lupus | Alaska | Recent |
| AMNH 1551 | Canis lupus | Oklahoma | Recent |
| AMNH 2247 | Canis lupus | Montana | Recent |
| AMNH 2384 | Canis lupus | Oklahoma | Recent |
| AMNH 4362 | Canis lupus | No data | Recent |
| AMNH 5378 | Canis lupus | Oklahoma | Recent |
| F:AM 30438 | Canis lupus - Beringian | Goldstream, AK | 45,500 ybp |
| F:AM 30439 | Canis lupus - Beringian | Head of Goldstream, AK | Rancholabrean |
| F:AM 30440 | Canis lupus - Beringian | Goldstream, AK | 38,000 ybp |
| F:AM 30474 | Canis lupus - Beringian | Goldstream, AK | Rancholabrean |
| F:AM 67168 | Canis lupus - Beringian | Engineer Creek, AK | 31,200 ybp |
| F:AM 67169 | Canis lupus - Beringian | Fairbanks Creek, AK | 20,305 ybp |
| F:AM 67198 | Canis lupus - Beringian | Engineer Creek, AK | Rancholabrean |
| F:AM 67208 | Canis lupus - Beringian | Lower Goldstream, AK | 41,040 ybp |
| F:AM 67224 | Canis lupus - Beringian | Fairbanks Creek, AK | 17,640 ybp |
| F:AM 67228 | Canis lupus - Beringian | Cripple Creek, AK | 32,100 ybp |
| F:AM 67230 | Canis lupus - Beringian | Cripple Creek, AK | Rancholabrean |
| F:AM 67235 | Canis lupus - Beringian | Cripple Creek, AK | 38,000 ybp |
| F:AM 67242 | Canis lupus - Beringian | Goldstream Banks at Fox, AK | Rancholabrean |
| F:AM 67245 | Canis lupus - Beringian | No. 2 Gold Strip, AK | Rancholabrean |
| F:AM 67250 | Canis lupus - Beringian | Ester Creek, AK | Rancholabrean |
| F:AM 67251 | Canis lupus - Beringian | Ester Creek, AK | Rancholabrean |
| F:AM 67253 | Canis lupus - Beringian | Cripple Creek, AK | Rancholabrean |
| F:AM 70943 | Canis lupus - Beringian | Gold Hill, AK | Rancholabrean |
| F:AM 70944 | Canis lupus - Beringian | Gold Hill, AK | 15,268 ybp |
| F:AM 70958 | Canis lupus - Beringian | Gold Hill, AK | 37,700 ybp |
| F:AM 97079 | Canis lupus - Beringian | Smith Gulch, Wiseman Arctic, AK | 45,500 ybp |
| KUVP 36054 | Canis sp. | Natural Trap Cave, WY | Rancholabrean |
| KUVP 43337 | Canis sp. | Natural Trap Cave, WY | Rancholabrean |
| KUVP 43811 | Canis sp. | Natural Trap Cave, WY | Rancholabrean |
| KUVP 45507 | Canis sp. | Natural Trap Cave, WY | Rancholabrean |
| KUVP 51276 | Canis sp. | Natural Trap Cave, WY | Rancholabrean |
| KUVP 57910 | Canis sp. | Natural Trap Cave, WY | Rancholabrean |
| KUVP 81602 | Canis sp. | Natural Trap Cave, WY | Rancholabrean |
| 2301-L-100 | Canis dirus | RLB pit 3 | ≈14-18 cal Kybp |
| 2301-L-102 | Canis dirus | RLB pit 3 | ≈14-18 cal Kybp |
| 2301-L-110 | Canis dirus | RLB pit 3 | ≈14-18 cal Kybp |
| 2301-L-111 | Canis dirus | RLB pit 3 | ≈14-18 cal Kybp |
| 2301-L-116 | Canis dirus | RLB pit 3 | ≈14-18 cal Kybp |
| 2301-L-118 | Canis dirus | RLB pit 3 | ≈14-18 cal Kybp |
| 2301-L-119 | Canis dirus | RLB pit 3 | ≈14-18 cal Kybp |
| 2301-L-122 | Canis dirus | RLB pit 3 | ≈14-18 cal Kybp |
| 2301-L-124 | Canis dirus | RLB pit 3 | ≈14-18 cal Kybp |
| 2301-L-125 | Canis dirus | RLB pit 3 | ≈14-18 cal Kybp |
| 2301-L-127 | Canis dirus | RLB pit 3 | ≈14-18 cal Kybp |
| 2301-L-128 | Canis dirus | RLB pit 3 | ≈14-18 cal Kybp |
| 2301-L-132 | Canis dirus | RLB pit 3 | ≈14-18 cal Kybp |
| 2301-L-133 | Canis dirus | RLB pit 3 | ≈14-18 cal Kybp |
| 2301-L-141 | Canis dirus | RLB pit 3 | ≈14-18 cal Kybp |
| 2301-L-144 | Canis dirus | RLB pit 3 | ≈14-18 cal Kybp |
| 2301-L-145 | Canis dirus | RLB pit 3 | ≈14-18 cal Kybp |
| 2301-L-149 | Canis dirus | RLB pit 3 | ≈14-18 cal Kybp |
| 2301-L-15 | Canis dirus | RLB pit 3 | ≈14-18 cal Kybp |
| 2301-L-153 | Canis dirus | RLB pit 3 | ≈14-18 cal Kybp |
| 2301-L-157 | Canis dirus | RLB pit 3 | ≈14-18 cal Kybp |
| 2301-L-162 | Canis dirus | RLB pit 3 | ≈14-18 cal Kybp |
| 2301-L-184 | Canis dirus | RLB pit 3 | ≈14-18 cal Kybp |
| 2301-L-189 | Canis dirus | RLB pit 3 | ≈14-18 cal Kybp |
| 2301-L-192 | Canis dirus | RLB pit 3 | ≈14-18 cal Kybp |
| 2301-L-207 | Canis dirus | RLB pit 3 | ≈14-18 cal Kybp |
| 2301-L-208 | Canis dirus | RLB pit 3 | ≈14-18 cal Kybp |
| 2301-L-209 | Canis dirus | RLB pit 3 | ≈14-18 cal Kybp |
| 2301-L-212 | Canis dirus | RLB pit 3 | ≈14-18 cal Kybp |
| 2301-L-214 | Canis dirus | RLB pit 3 | ≈14-18 cal Kybp |
| 2301-L-215 | Canis dirus | RLB pit 3 | ≈14-18 cal Kybp |
| 2301-L-216 | Canis dirus | RLB pit 3 | ≈14-18 cal Kybp |
| 2301-L-217 | Canis dirus | RLB pit 3 | ≈14-18 cal Kybp |
| 2301-L-220 | Canis dirus | RLB pit 3 | ≈14-18 cal Kybp |
| 2301-L-224 | Canis dirus | RLB pit 3 | ≈14-18 cal Kybp |
| 2301-L-403 | Canis dirus | RLB pit 3 | ≈14-18 cal Kybp |
| 2301-L-412 | Canis dirus | RLB pit 3 | ≈14-18 cal Kybp |
| 2301-L-416 | Canis dirus | RLB pit 3 | ≈14-18 cal Kybp |
| 2301-L-426 | Canis dirus | RLB pit 3 | ≈14-18 cal Kybp |
| 2301-L-436 | Canis dirus | RLB pit 3 | ≈14-18 cal Kybp |
| 2301-L-445 | Canis dirus | RLB pit 3 | ≈14-18 cal Kybp |
| 2301-L-458 | Canis dirus | RLB pit 3 | ≈14-18 cal Kybp |
| 2301-L-459 | Canis dirus | RLB pit 3 | ≈14-18 cal Kybp |
| 2301-L-463 | Canis dirus | RLB pit 3 | ≈14-18 cal Kybp |
| 2301-L-464 | Canis dirus | RLB pit 3 | ≈14-18 cal Kybp |
| 2301-L-472 | Canis dirus | RLB pit 3 | ≈14-18 cal Kybp |
| 2301-L-473 | Canis dirus | RLB pit 3 | ≈14-18 cal Kybp |
| 2301-L-477 | Canis dirus | RLB pit 3 | ≈14-18 cal Kybp |
| 2301-L-478 | Canis dirus | RLB pit 3 | ≈14-18 cal Kybp |
| 2301-L-484 | Canis dirus | RLB pit 3 | ≈14-18 cal Kybp |
| 2301-L-486 | Canis dirus | RLB pit 3 | ≈14-18 cal Kybp |
| 2301-L-488 | Canis dirus | RLB pit 3 | ≈14-18 cal Kybp |
| 2301-L-489 | Canis dirus | RLB pit 3 | ≈14-18 cal Kybp |
| 2301-L-491 | Canis dirus | RLB pit 3 | ≈14-18 cal Kybp |
| 2301-L-502 | Canis dirus | RLB pit 3 | ≈14-18 cal Kybp |
| 2301-L-505 | Canis dirus | RLB pit 3 | ≈14-18 cal Kybp |
| 2301-L-52 | Canis dirus | RLB pit 3 | ≈14-18 cal Kybp |
| 2301-L-59 | Canis dirus | RLB pit 3 | ≈14-18 cal Kybp |
| 2301-L-61 | Canis dirus | RLB pit 3 | ≈14-18 cal Kybp |
| 2301-L-62 | Canis dirus | RLB pit 3 | ≈14-18 cal Kybp |
| 2301-L-71 | Canis dirus | RLB pit 3 | ≈14-18 cal Kybp |
| 2301-L-77 | Canis dirus | RLB pit 3 | ≈14-18 cal Kybp |
| 2301-L-82 | Canis dirus | RLB pit 3 | ≈14-18 cal Kybp |
| 2301-L-91 | Canis dirus | RLB pit 3 | ≈14-18 cal Kybp |
| 2301-R-205 | Canis dirus | RLB pit 3 | ≈14-18 cal Kybp |
| 2301-R-225 | Canis dirus | RLB pit 3 | ≈14-18 cal Kybp |
| LACMHC 53832 | Canis dirus | RLB pit 3 | ≈14-18 cal Kybp |
| LACMHC 53833 | Canis dirus | RLB pit 3 | ≈14-18 cal Kybp |
| LACMHC 53843 | Canis dirus | RLB pit 3 | ≈14-18 cal Kybp |
| LACMHC 53845 | Canis dirus | RLB pit 3 | ≈14-18 cal Kybp |
| LACMHC 53846 | Canis dirus | RLB pit 3 | ≈14-18 cal Kybp |
| LACMHC 53852 | Canis dirus | RLB pit 3 | ≈14-18 cal Kybp |
| LACMHC 53853 | Canis dirus | RLB pit 3 | ≈14-18 cal Kybp |
| LACMHC 53854 | Canis dirus | RLB pit 3 | ≈14-18 cal Kybp |
| LACMHC 53855 | Canis dirus | RLB pit 3 | ≈14-18 cal Kybp |
| LACMHC 53857 | Canis dirus | RLB pit 3 | ≈14-18 cal Kybp |
| LACMHC 53858 | Canis dirus | RLB pit 3 | ≈14-18 cal Kybp |
| 2301-L-105 | Canis dirus | RLB pit 4 | ≈14-16 cal Kybp |
| 2301-L-107 | Canis dirus | RLB pit 4 | ≈14-16 cal Kybp |
| 2301-L-117 | Canis dirus | RLB pit 4 | ≈14-16 cal Kybp |
| 2301-L-121 | Canis dirus | RLB pit 4 | ≈14-16 cal Kybp |
| 2301-L-129 | Canis dirus | RLB pit 4 | ≈14-16 cal Kybp |
| 2301-L-135 | Canis dirus | RLB pit 4 | ≈14-16 cal Kybp |
| 2301-L-136 | Canis dirus | RLB pit 4 | ≈14-16 cal Kybp |
| 2301-L-137 | Canis dirus | RLB pit 4 | ≈14-16 cal Kybp |
| 2301-L-138 | Canis dirus | RLB pit 4 | ≈14-16 cal Kybp |
| 2301-L-142 | Canis dirus | RLB pit 4 | ≈14-16 cal Kybp |
| 2301-L-146 | Canis dirus | RLB pit 4 | ≈14-16 cal Kybp |
| 2301-L-152 | Canis dirus | RLB pit 4 | ≈14-16 cal Kybp |
| 2301-L-158 | Canis dirus | RLB pit 4 | ≈14-16 cal Kybp |
| 2301-L-160 | Canis dirus | RLB pit 4 | ≈14-16 cal Kybp |
| 2301-L-161 | Canis dirus | RLB pit 4 | ≈14-16 cal Kybp |
| 2301-L-165 | Canis dirus | RLB pit 4 | ≈14-16 cal Kybp |
| 2301-L-166 | Canis dirus | RLB pit 4 | ≈14-16 cal Kybp |
| 2301-L-168 | Canis dirus | RLB pit 4 | ≈14-16 cal Kybp |
| 2301-L-176 | Canis dirus | RLB pit 4 | ≈14-16 cal Kybp |
| 2301-L-183 | Canis dirus | RLB pit 4 | ≈14-16 cal Kybp |
| 2301-L-187 | Canis dirus | RLB pit 4 | ≈14-16 cal Kybp |
| 2301-L-194 | Canis dirus | RLB pit 4 | ≈14-16 cal Kybp |
| 2301-L-200 | Canis dirus | RLB pit 4 | ≈14-16 cal Kybp |
| 2301-L-378 | Canis dirus | RLB pit 4 | ≈14-16 cal Kybp |
| 2301-L-387 | Canis dirus | RLB pit 4 | ≈14-16 cal Kybp |
| 2301-L-415 | Canis dirus | RLB pit 4 | ≈14-16 cal Kybp |
| 2301-L-418 | Canis dirus | RLB pit 4 | ≈14-16 cal Kybp |
| 2301-L-434 | Canis dirus | RLB pit 4 | ≈14-16 cal Kybp |
| 2301-L-479 | Canis dirus | RLB pit 4 | ≈14-16 cal Kybp |
| 2301-L-496 | Canis dirus | RLB pit 4 | ≈14-16 cal Kybp |
| 2301-L-504 | Canis dirus | RLB pit 4 | ≈14-16 cal Kybp |
| 2301-L-75 | Canis dirus | RLB pit 4 | ≈14-16 cal Kybp |
| 2301-L-76 | Canis dirus | RLB pit 4 | ≈14-16 cal Kybp |
| 2301-L-96 | Canis dirus | RLB pit 4 | ≈14-16 cal Kybp |
| 2301-R-250 | Canis dirus | RLB pit 4 | ≈14-16 cal Kybp |
| LACMHC 54622 | Canis dirus | RLB pit 4 | ≈14-16 cal Kybp |
| LACMHC 54627 | Canis dirus | RLB pit 4 | ≈14-16 cal Kybp |
| LACMHC 54640 | Canis dirus | RLB pit 4 | ≈14-16 cal Kybp |
| LACMHC 54662 | Canis dirus | RLB pit 4 | ≈14-16 cal Kybp |
| LACMHC 54663 | Canis dirus | RLB pit 4 | ≈14-16 cal Kybp |
| 2301-L-101 | Canis dirus | RLB pit 13 | ≈17-18 cal Kybp |
| 2301-L-143 | Canis dirus | RLB pit 13 | ≈17-18 cal Kybp |
| 2301-L-173 | Canis dirus | RLB pit 13 | ≈17-18 cal Kybp |
| 2301-L-419 | Canis dirus | RLB pit 13 | ≈17-18 cal Kybp |
| 2301-L-447 | Canis dirus | RLB pit 13 | ≈17-18 cal Kybp |
| 2301-L-451 | Canis dirus | RLB pit 13 | ≈17-18 cal Kybp |
| 2301-L-466 | Canis dirus | RLB pit 13 | ≈17-18 cal Kybp |
| 2301-L-468 | Canis dirus | RLB pit 13 | ≈17-18 cal Kybp |
| 2301-L-474 | Canis dirus | RLB pit 13 | ≈17-18 cal Kybp |
| 2301-L-475 | Canis dirus | RLB pit 13 | ≈17-18 cal Kybp |
| 2301-L-490 | Canis dirus | RLB pit 13 | ≈17-18 cal Kybp |
| 2301-L-500 | Canis dirus | RLB pit 13 | ≈17-18 cal Kybp |
| 2301-L-63 | Canis dirus | RLB pit 13 | ≈17-18 cal Kybp |
| 2301-L-70 | Canis dirus | RLB pit 13 | ≈17-18 cal Kybp |
| 2301-L-72 | Canis dirus | RLB pit 13 | ≈17-18 cal Kybp |
| 2301-L-92 | Canis dirus | RLB pit 13 | ≈17-18 cal Kybp |
| 2301-L-93 | Canis dirus | RLB pit 13 | ≈17-18 cal Kybp |
| LACMHC 54899 | Canis dirus | RLB pit 13 | ≈17-18 cal Kybp |
| LACMHC 54901 | Canis dirus | RLB pit 13 | ≈17-18 cal Kybp |
| LACMHC 54903 | Canis dirus | RLB pit 13 | ≈17-18 cal Kybp |
| LACMHC 54909 | Canis dirus | RLB pit 13 | ≈17-18 cal Kybp |
| LACMHC 54912 | Canis dirus | RLB pit 13 | ≈17-18 cal Kybp |
| LACMHC 54913 | Canis dirus | RLB pit 13 | ≈17-18 cal Kybp |
| LACMHC 54917 | Canis dirus | RLB pit 13 | ≈17-18 cal Kybp |
| LACMHC 54921 | Canis dirus | RLB pit 13 | ≈17-18 cal Kybp |
| LACMHC 54922 | Canis dirus | RLB pit 13 | ≈17-18 cal Kybp |
| LACMHC 54924 | Canis dirus | RLB pit 13 | ≈17-18 cal Kybp |
| LACMHC 54940 | Canis dirus | RLB pit 13 | ≈17-18 cal Kybp |
| LACMHC 54943 | Canis dirus | RLB pit 13 | ≈17-18 cal Kybp |
| LACMHC 54958 | Canis dirus | RLB pit 13 | ≈17-18 cal Kybp |
| LACMHC 54959 | Canis dirus | RLB pit 13 | ≈17-18 cal Kybp |
| LACMHC 54962 | Canis dirus | RLB pit 13 | ≈17-18 cal Kybp |
| LACMHC 54975 | Canis dirus | RLB pit 13 | ≈17-18 cal Kybp |
| LACMHC 54976 | Canis dirus | RLB pit 13 | ≈17-18 cal Kybp |
| LACMHC 54977 | Canis dirus | RLB pit 13 | ≈17-18 cal Kybp |
| LACMHC 54978 | Canis dirus | RLB pit 13 | ≈17-18 cal Kybp |
| LACMHC 54983 | Canis dirus | RLB pit 13 | ≈17-18 cal Kybp |
| 2301-L-375 | Canis dirus | RLB pit 77 | ≈32-37 cal Kybp |
| 2301-L-437 | Canis dirus | RLB pit 77 | ≈32-37 cal Kybp |
| 2301-L-462 | Canis dirus | RLB pit 77 | ≈32-37 cal Kybp |
| 2301-L-467 | Canis dirus | RLB pit 77 | ≈32-37 cal Kybp |
| LACMHC 56078 | Canis dirus | RLB pit 77 | ≈32-37 cal Kybp |
| LACMHC 56079 | Canis dirus | RLB pit 77 | ≈32-37 cal Kybp |
| R12527 | Canis dirus | RLB pit 91 | ≈25-28 cal Kybp |
| R17460 | Canis dirus | RLB pit 91 | ≈25-28 cal Kybp |
| R17488 | Canis dirus | RLB pit 91 | ≈25-28 cal Kybp |
| R17928 | Canis dirus | RLB pit 91 | ≈25-28 cal Kybp |
| R18167 | Canis dirus | RLB pit 91 | ≈25-28 cal Kybp |
| R18664 | Canis dirus | RLB pit 91 | ≈25-28 cal Kybp |
| R23473 | Canis dirus | RLB pit 91 | ≈25-28 cal Kybp |
| R26151 | Canis dirus | RLB pit 91 | ≈25-28 cal Kybp |
| R27246 | Canis dirus | RLB pit 91 | ≈25-28 cal Kybp |
| R27807 | Canis dirus | RLB pit 91 | ≈25-28 cal Kybp |
| R28351 | Canis dirus | RLB pit 91 | ≈25-28 cal Kybp |
| R28379 | Canis dirus | RLB pit 91 | ≈25-28 cal Kybp |
| R28913 | Canis dirus | RLB pit 91 | ≈25-28 cal Kybp |
| R28915 | Canis dirus | RLB pit 91 | ≈25-28 cal Kybp |
| R30351 | Canis dirus | RLB pit 91 | ≈25-28 cal Kybp |
| R30721 | Canis dirus | RLB pit 91 | ≈25-28 cal Kybp |
| R30746 | Canis dirus | RLB pit 91 | ≈25-28 cal Kybp |
| R31305 | Canis dirus | RLB pit 91 | ≈25-28 cal Kybp |
| R34526 | Canis dirus | RLB pit 91 | ≈25-28 cal Kybp |
| R34706 | Canis dirus | RLB pit 91 | ≈25-28 cal Kybp |
| R37298 | Canis dirus | RLB pit 91 | ≈25-28 cal Kybp |
| R38491 | Canis dirus | RLB pit 91 | ≈25-28 cal Kybp |
| R39009 | Canis dirus | RLB pit 91 | ≈25-28 cal Kybp |
| R39106 | Canis dirus | RLB pit 91 | ≈25-28 cal Kybp |
| R39216 | Canis dirus | RLB pit 91 | ≈25-28 cal Kybp |
| R39376 | Canis dirus | RLB pit 91 | ≈25-28 cal Kybp |
| R39566 | Canis dirus | RLB pit 91 | ≈25-28 cal Kybp |
| R39848 | Canis dirus | RLB pit 91 | ≈25-28 cal Kybp |
| R39965 | Canis dirus | RLB pit 91 | ≈25-28 cal Kybp |
| R40942 | Canis dirus | RLB pit 91 | ≈25-28 cal Kybp |
| R41696 | Canis dirus | RLB pit 91 | ≈25-28 cal Kybp |
| R41748 | Canis dirus | RLB pit 91 | ≈25-28 cal Kybp |
| R43672 | Canis dirus | RLB pit 91 | ≈25-28 cal Kybp |
| R46274 | Canis dirus | RLB pit 91 | ≈25-28 cal Kybp |
| R49189 | Canis dirus | RLB pit 91 | ≈25-28 cal Kybp |
| R52965 | Canis dirus | RLB pit 91 | ≈25-28 cal Kybp |
| R52966 | Canis dirus | RLB pit 91 | ≈25-28 cal Kybp |
| R53460 | Canis dirus | RLB pit 91 | ≈25-28 cal Kybp |
| R53605 | Canis dirus | RLB pit 91 | ≈25-28 cal Kybp |
| R53878 | Canis dirus | RLB pit 91 | ≈25-28 cal Kybp |
| 28654 | Canis dirus | RLB pit 2051 | ≈21-30 cal Kybp |
| 28662 | Canis dirus | RLB pit 2051 | ≈21-30 cal Kybp |
| 28663 | Canis dirus | RLB pit 2051 | ≈21-30 cal Kybp |
| 28668 | Canis dirus | RLB pit 2051 | ≈21-30 cal Kybp |
| 28685 | Canis dirus | RLB pit 2051 | ≈21-30 cal Kybp |
| 28686 | Canis dirus | RLB pit 2051 | ≈21-30 cal Kybp |
| 28687 | Canis dirus | RLB pit 2051 | ≈21-30 cal Kybp |
| 28698 | Canis dirus | RLB pit 2051 | ≈21-30 cal Kybp |
| 28720 | Canis dirus | RLB pit 2051 | ≈21-30 cal Kybp |
| 28736 | Canis dirus | RLB pit 2051 | ≈21-30 cal Kybp |
| 28738 | Canis dirus | RLB pit 2051 | ≈21-30 cal Kybp |
| 28739 | Canis dirus | RLB pit 2051 | ≈21-30 cal Kybp |
| 28750 | Canis dirus | RLB pit 2051 | ≈21-30 cal Kybp |
| 28756 | Canis dirus | RLB pit 2051 | ≈21-30 cal Kybp |
| 28758 | Canis dirus | RLB pit 2051 | ≈21-30 cal Kybp |
| 29041 | Canis dirus | RLB pit 2051 | ≈21-30 cal Kybp |
| 29049 | Canis dirus | RLB pit 2051 | ≈21-30 cal Kybp |
| 29056 | Canis dirus | RLB pit 2051 | ≈21-30 cal Kybp |
| 29066 | Canis dirus | RLB pit 2051 | ≈21-30 cal Kybp |
| 29068 | Canis dirus | RLB pit 2051 | ≈21-30 cal Kybp |
| 29075 | Canis dirus | RLB pit 2051 | ≈21-30 cal Kybp |
| 29082 | Canis dirus | RLB pit 2051 | ≈21-30 cal Kybp |
| 76919 | Canis dirus | RLB pit 2051 | ≈21-30 cal Kybp |
| 28738b | Canis dirus | RLB pit 2051 | ≈21-30 cal Kybp |
| 28739b | Canis dirus | RLB pit 2051 | ≈21-30 cal Kybp |
| 29075b | Canis dirus | RLB pit 2051 | ≈21-30 cal Kybp |
| 2301-L-10 | Canis dirus | RLB pit 61-67 | ≈13-14 cal Kybp |
| 2301-L-106 | Canis dirus | RLB pit 61-67 | ≈13-14 cal Kybp |
| 2301-L-109 | Canis dirus | RLB pit 61-67 | ≈13-14 cal Kybp |
| 2301-L-12 | Canis dirus | RLB pit 61-67 | ≈13-14 cal Kybp |
| 2301-L-120 | Canis dirus | RLB pit 61-67 | ≈13-14 cal Kybp |
| 2301-L-123 | Canis dirus | RLB pit 61-67 | ≈13-14 cal Kybp |
| 2301-L-140 | Canis dirus | RLB pit 61-67 | ≈13-14 cal Kybp |
| 2301-L-148 | Canis dirus | RLB pit 61-67 | ≈13-14 cal Kybp |
| 2301-L-154 | Canis dirus | RLB pit 61-67 | ≈13-14 cal Kybp |
| 2301-L-155 | Canis dirus | RLB pit 61-67 | ≈13-14 cal Kybp |
| 2301-L-156 | Canis dirus | RLB pit 61-67 | ≈13-14 cal Kybp |
| 2301-L-164 | Canis dirus | RLB pit 61-67 | ≈13-14 cal Kybp |
| 2301-L-167 | Canis dirus | RLB pit 61-67 | ≈13-14 cal Kybp |
| 2301-L-185 | Canis dirus | RLB pit 61-67 | ≈13-14 cal Kybp |
| 2301-L-186 | Canis dirus | RLB pit 61-67 | ≈13-14 cal Kybp |
| 2301-L-193 | Canis dirus | RLB pit 61-67 | ≈13-14 cal Kybp |
| 2301-L-203 | Canis dirus | RLB pit 61-67 | ≈13-14 cal Kybp |
| 2301-L-210 | Canis dirus | RLB pit 61-67 | ≈13-14 cal Kybp |
| 2301-L-211 | Canis dirus | RLB pit 61-67 | ≈13-14 cal Kybp |
| 2301-L-218 | Canis dirus | RLB pit 61-67 | ≈13-14 cal Kybp |
| 2301-L-242 | Canis dirus | RLB pit 61-67 | ≈13-14 cal Kybp |
| 2301-L-244 | Canis dirus | RLB pit 61-67 | ≈13-14 cal Kybp |
| 2301-L-245 | Canis dirus | RLB pit 61-67 | ≈13-14 cal Kybp |
| 2301-L-246 | Canis dirus | RLB pit 61-67 | ≈13-14 cal Kybp |
| 2301-L-28 | Canis dirus | RLB pit 61-67 | ≈13-14 cal Kybp |
| 2301-L-4 | Canis dirus | RLB pit 61-67 | ≈13-14 cal Kybp |
| 2301-L-42 | Canis dirus | RLB pit 61-67 | ≈13-14 cal Kybp |
| 2301-L-425 | Canis dirus | RLB pit 61-67 | ≈13-14 cal Kybp |
| 2301-L-428 | Canis dirus | RLB pit 61-67 | ≈13-14 cal Kybp |
| 2301-L-430 | Canis dirus | RLB pit 61-67 | ≈13-14 cal Kybp |
| 2301-L-433 | Canis dirus | RLB pit 61-67 | ≈13-14 cal Kybp |
| 2301-L-44 | Canis dirus | RLB pit 61-67 | ≈13-14 cal Kybp |
| 2301-L-455 | Canis dirus | RLB pit 61-67 | ≈13-14 cal Kybp |
| 2301-L-469 | Canis dirus | RLB pit 61-67 | ≈13-14 cal Kybp |
| 2301-L-48 | Canis dirus | RLB pit 61-67 | ≈13-14 cal Kybp |
| 2301-L-482 | Canis dirus | RLB pit 61-67 | ≈13-14 cal Kybp |
| 2301-L-493 | Canis dirus | RLB pit 61-67 | ≈13-14 cal Kybp |
| 2301-L-50 | Canis dirus | RLB pit 61-67 | ≈13-14 cal Kybp |
| 2301-L-509 | Canis dirus | RLB pit 61-67 | ≈13-14 cal Kybp |
| 2301-L-511 | Canis dirus | RLB pit 61-67 | ≈13-14 cal Kybp |
| 2301-L-514 | Canis dirus | RLB pit 61-67 | ≈13-14 cal Kybp |
| 2301-L-516 | Canis dirus | RLB pit 61-67 | ≈13-14 cal Kybp |
| 2301-L-517 | Canis dirus | RLB pit 61-67 | ≈13-14 cal Kybp |
| 2301-L-518 | Canis dirus | RLB pit 61-67 | ≈13-14 cal Kybp |
| 2301-L-537 | Canis dirus | RLB pit 61-67 | ≈13-14 cal Kybp |
| 2301-L-64 | Canis dirus | RLB pit 61-67 | ≈13-14 cal Kybp |
| 2301-L-65 | Canis dirus | RLB pit 61-67 | ≈13-14 cal Kybp |
| 2301-L-66 | Canis dirus | RLB pit 61-67 | ≈13-14 cal Kybp |
| 2301-L-68 | Canis dirus | RLB pit 61-67 | ≈13-14 cal Kybp |
| 2301-L-74 | Canis dirus | RLB pit 61-67 | ≈13-14 cal Kybp |
| 2301-L-79 | Canis dirus | RLB pit 61-67 | ≈13-14 cal Kybp |
| 2301-L-81 | Canis dirus | RLB pit 61-67 | ≈13-14 cal Kybp |
| 2301-L-84 | Canis dirus | RLB pit 61-67 | ≈13-14 cal Kybp |
| 2301-L-85 | Canis dirus | RLB pit 61-67 | ≈13-14 cal Kybp |
| 2301-L-95 | Canis dirus | RLB pit 61-67 | ≈13-14 cal Kybp |
| 2301-L-97 | Canis dirus | RLB pit 61-67 | ≈13-14 cal Kybp |
| 2301-L-99 | Canis dirus | RLB pit 61-67 | ≈13-14 cal Kybp |
| LACMHC 55509 | Canis dirus | RLB pit 61-67 | ≈13-14 cal Kybp |
| LACMHC 55513 | Canis dirus | RLB pit 61-67 | ≈13-14 cal Kybp |
| LACMHC 55516 | Canis dirus | RLB pit 61-67 | ≈13-14 cal Kybp |
| LACMHC 55518 | Canis dirus | RLB pit 61-67 | ≈13-14 cal Kybp |
| LACMHC 55520 | Canis dirus | RLB pit 61-67 | ≈13-14 cal Kybp |
| LACMHC 55521 | Canis dirus | RLB pit 61-67 | ≈13-14 cal Kybp |
| LACMHC 55660 | Canis dirus | RLB pit 61-67 | ≈13-14 cal Kybp |
| LACMHC 55665 | Canis dirus | RLB pit 61-67 | ≈13-14 cal Kybp |
| LACMHC 55666 | Canis dirus | RLB pit 61-67 | ≈13-14 cal Kybp |
| LACMHC 55667 | Canis dirus | RLB pit 61-67 | ≈13-14 cal Kybp |
| LACMHC 55675 | Canis dirus | RLB pit 61-67 | ≈13-14 cal Kybp |
| LACMHC 55681 | Canis dirus | RLB pit 61-67 | ≈13-14 cal Kybp |
| LACMHC 55683 | Canis dirus | RLB pit 61-67 | ≈13-14 cal Kybp |
| LACMHC 55685 | Canis dirus | RLB pit 61-67 | ≈13-14 cal Kybp |
| LACMHC 55691 | Canis dirus | RLB pit 61-67 | ≈13-14 cal Kybp |
| LACMHC 55692 | Canis dirus | RLB pit 61-67 | ≈13-14 cal Kybp |
| LACMHC 55693 | Canis dirus | RLB pit 61-67 | ≈13-14 cal Kybp |
| LACMHC 55694 | Canis dirus | RLB pit 61-67 | ≈13-14 cal Kybp |
| LACMHC 55695 | Canis dirus | RLB pit 61-67 | ≈13-14 cal Kybp |
| LACMHC 675 | Canis dirus | RLB pit 61-67 | ≈13-14 cal Kybp |
| LACMHC 676 | Canis dirus | RLB pit 61-67 | ≈13-14 cal Kybp |
| LACMHC 680 | Canis dirus | RLB pit 61-67 | ≈13-14 cal Kybp |
| LACMHC 681 | Canis dirus | RLB pit 61-67 | ≈13-14 cal Kybp |
| LACMHC 682 | Canis dirus | RLB pit 61-67 | ≈13-14 cal Kybp |
| LACMHC 683 | Canis dirus | RLB pit 61-67 | ≈13-14 cal Kybp |
